# Atomic Layer-Deposited Silane Coupling Agent for Interface Passivation of Quantum Dot Light-Emitting Diodes

**DOI:** 10.1021/acs.jpclett.4c01974

**Published:** 2024-09-03

**Authors:** Ting Ding, Yin-Man Song, Meng-Wei Wang, Hang Liu, Jing Jiang, Jin-Cheng Xu, Hong-Chao Liu, Kar-Wei Ng, Shuang-Peng Wang

**Affiliations:** Institute of Applied Physics and Materials Engineering, University of Macau, Taipa, Macao SAR 999078, China

## Abstract

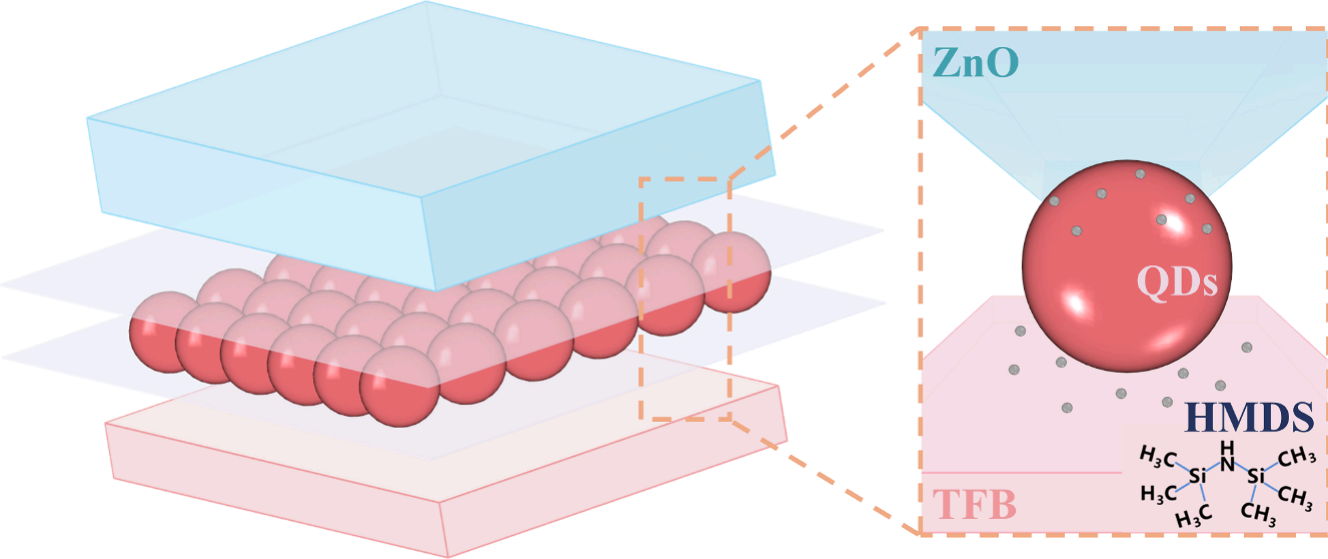

Inserting an insulating layer between the charge transport layer (CTL) and quantum dot emitting layer (QDL) is widely used in improving the performance of quantum dot light-emitting diodes (QLEDs). However, the additional layer inevitably leads to energy loss and joule heat. Herein, a monolayer silane coupling agent is used to modify the said interfaces via the self-limiting adsorption effect. Because the ultrathin layers induce negligible series resistance to the device, they can partially passivate the interfacial defects on the electron transport side and help confine the electrons within the QDL on the hole transport side. These interfacial modifications can not only suppress the nonradiative recombination but also slow down the aging of the hole transport layer. The findings here underline a low-temperature adsorption-based strategy for effective interfacial modification which can be used in any layer-by-layer device structures.

Solution-processed quantum dot light-emitting diodes (QLEDs) are considered a promising candidate for the next generation display technology due to their high color purity, wide color gamut, and low power consumption.^1−3^ After decades of development, the external quantum efficiency (EQE) of red, green, and blue QLEDs has surpassed 20%,^4−6^ which is comparable to that of organic light-emitting diodes (OLEDs), showing significant progress toward industrialization. Currently, most QLEDs adopt a sandwich structure,^7,8^ while unexpected interface states are introduced inevitably between the functional layers during the layer-by-layer stacking process. It has been experimentally proven that the imperfect heterostructure interface can lead to additional potential barriers^9^ and nonradiative recombination centers,^10−12^ affecting the carrier transport and recombination process. The interface modulation between the charge functional layer and the QD emission layer has a significant impact on and determines the device performances. In 2014, with the help of a thin PMMA insulating layer, Dai et al.^13^ achieved the first QLED with EQE exceeding 20% and greatly improved the device’s operation lifetime. Other studies have also confirmed that inserting a charge isolation layer, such as PEI,^14^ Al_2_O_3_,^15^ PEIE,^16^ and MgO,^17^ between the QD emission layer and the charge functional layers can effectively reduce nonradiative recombination, enhance the illumination performance, and minimize the positive aging effect of QLEDs.^18^ The isolation layer can effectively confine the carrier recombination region and effectively passivate the charge transport layer. However, the presence of the charge isolation layer inevitably leads to energy loss of carriers and the accumulation of Joule heat under a high injection current. Therefore, striking a balance between the energy loss and passivation effect becomes a challenge in this approach.

Herein, drawing inspiration from atomic layer deposition (ALD), an ultrathin single-layer silane coupling agent is used to passivate the interface between the QD layer and charge transport layer (CTL), which is proven to be effective in suppressing leakage current at the CTL/QD interface. By placing the charge transport layer in an atmosphere of silane coupling agent (Bis(trimethylsilyl)amine, HMDS), a dense monolayer of HMDS forms at the CTL/QD interface due to the self-limiting adsorption effect. The ultrathin monolayer not only induces negligible series resistance to the entire device structure, but also noticeably enhances the current efficiency and lifetime of the QLED. Detailed electrical and material characterizations reveal that the HMDS layer at the hole transport layer (HTL)/QD interface can effectively block the overflow of electrons into the HTL, thus reducing the leakage current and charge accumulation at the HTL. At the electron transport layer (ETL)/QD interface, the monolayer passivates the interface defects of the ZnO layer and suppresses the nonradiative recombination. The combination of these effects leads to an 8% enhancement in the device current efficiency and a 50% boost in operation lifetime. Similar improvements can also be observed when we replace HMDS with other silane coupling agents, attesting to the versatility of this adsorption-based technique. The findings here underline a low-temperature adsorption-based strategy for the effective modification of interfaces in not only QLED structures, but also any device architecture with low fabrication thermal budget.

As the basis of ALD, the self-limiting adsorption is the key to achieving a uniform monolayer. As shown in Figure 1a, to replicate the ALD procedure, the substrates coated with different underlying layers are placed in an airtight container along with a droplet of HMDS. When the container is heated (90 °C here), the HMDS vaporizes, and the molecules are evenly distributed in the container. After tens of seconds of treatment, a monolayer HMDS is formed on the surface of the samples due to the self-limiting adsorption effect.^19^ During this processing period, the vapor phase pressure of HMDS maintains at its saturated vapor pressure, as confirmed by the observation of liquefied HMDS solvent at the top of the container. The conventional bottom-emitting QLED structure (Glass/ITO/PEDOT:PSS/TFB/QDs/ZnO/Al) is used in this study, and the energy band alignment of the QLED is shown in Figure 1b. All the fabrication details can be found in Supporting Information. The HMDS atmosphere modification occurred at both the HTL/QDs and QDs/ETL interfaces. The insertion layer does not lead to obvious changes in the morphology of the subsequent functional layers. (Figures S1 and S2). The cross-sectional high-resolution transmission electron microscopy (HR-TEM) images (Figure S3) confirm that the insertion of HMDS has unnoticeable effect on the device structure. The energy-dispersive spectrometer (EDS) line-scan profiles (Figure 1c) show that the signal of Si observed is remarkably faint, implying the presence of silicon in the processed device is negligible. This suggests that the HMDS absorption layer is exceptionally thin, thus hinting that it has low impedance to carrier transportation.

**Figure 1:**
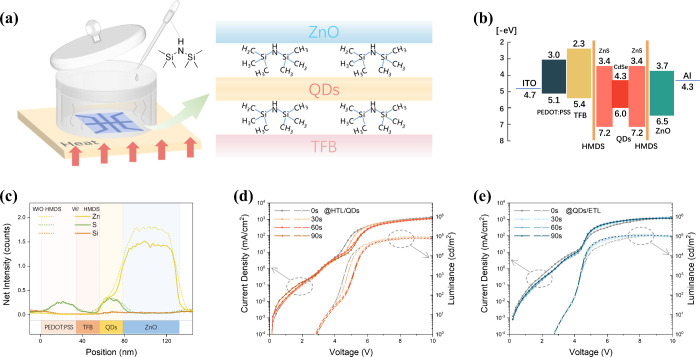
Interfacial modification for QLEDs based on CdSe/ZnS QDs via the self-limiting adsorption of silane coupling agent molecules. **a**, Schematic diagram illustrating the deposition of bis(trimethylsilyl)amine (HMDS) at the two CTL/QD layer interfaces. We put the underlying films in an airtight container, which is filled with HMDS atmosphere by dropping the HMDS liquid in container heated at 90 °C. **b**, Energy band alignment of the QLED structure used in this work. **c**, Line profiles of Zn, S and Si for the cross-section of devices with and without HMDS modification obtained with cross-sectional TEM-EDS. Current density versus voltage curves (left) and luminance versus voltage curves (right) of **d**, HTL/HMDS/QDs devices and **e**, QDs/HMDS/ETL devices under different HMDS exposure times.

However, when HMDS insertions are performed on the HTL/QDs interface and the QDs/ETL interface with different processing durations, the devices’ current density–voltage-luminance (J-V-L) characteristics exhibit obvious differences. As shown in Figure 1d, modifying the HTL/QDs interface with HMDS alone leads to a decrease in both current density and luminance, and the effect becomes more prominent with increasing processing time. These reductions in current density and luminance, particularly pronounced when the device bias is in between 4 and 6 V, may originate from the series resistance introduced by the insulating nature of HMDS. However, no significant difference was observed for processing times of 60 and 90 s. This indicates that the HMDS adsorption reaches saturation at 60 s such that a dense monolayer forms, in line with the ALD self-adsorption limitation principle. The deduction above is confirmed by the J-V-L curves of the samples with only the QDs/ETL interface modified with HMDS. As shown in Figure 1e, both luminance and current density change with the processing time and are nearly identical for the 60 and 90 s samples, while the performance is correlated with processing time before 60 s. However, in contrast to the HTL/QDs interface, when HMDS is inserted into the QDs/ETL interface, there is an increase in both current density and luminance. The above observations show that the monolayer HMDS can effectively affect the carrier transport and recombination in QLED, but its effect on the HTL/QDs and QDs/ETL interfaces differs significantly.

To verify the function of HMDS insertion, we compare the performances of devices with HMDS inserted at different interfaces. When the HMDS is applied at the HTL/QDs interface (see the red and indigo curves in Figure 2a), the current density decreases after turn-on due to the insulating nature of the HMDS silane coupling agent. Meanwhile, it shows that the turn-on voltage of the two samples is obviously higher than that of the devices without HTL modification, which confirms that the HMDS insertion at the HTL side can lead to an increased series resistance of the device.^20^ The hole-only device offers direct evidence (Figure S4), showing that the current density is significantly reduced by the insertion of HMDS. It is noticeable that the device’s current efficiency (CE) becomes slightly higher in devices with HTL modifications (Figure 2b). The improvement can be attributed to HMDS preventing excess electrons from entering the HTL (Figure S5), while the leakage current would be a primary cause of the HTL degradation.^21^

**Figure 2:**
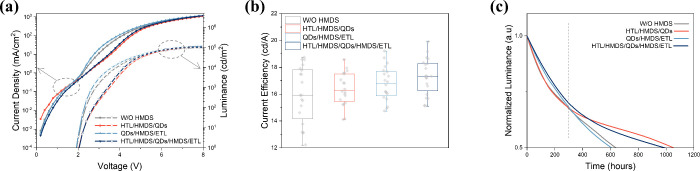
Electrical and aging tests on four groups of devices with different interfacial modifications. **a**, Current density versus voltage curves (left) and luminance versus voltage curves (right). **b**, Box plots of current efficiency obtained from twenty sets of devices. **c**, Aging test for four groups of devices under a constant current density, whose initial brightness is about 10000 cd/m^2^. The lifetime of devices is simulated at initial brightness of 1000 cd/m^2^ based on the relation *L*_0_^*n*^ × *T*_50_ = *C*. (*n* = 1.8)

In contrast, when the HMDS insertion is performed at the QDs/ETL interface, the current density and luminance increase slightly compared to the control device (gray curve in Figure 2a), while the turn-on voltage remains unchanged (sky-blue curve Figure 2a), and the CE is also increased (Figure 2b). Similar phenomena can also be observed when HMDS is inserted at the QDs/ETL interface in the devices with HMDS-coated HTL (Figure 2a, indigo compared to red curves). This suggests that the HMDS on the ETL side barely hinders the carrier transport and can slightly suppress nonradiative recombination near the interface concerned. This performance improvement may stem from the effective passivation of the surface defects in the ZnO layer (Figure S6).

To validate the effectiveness of HMDS insertion in improving QLED performance, 20 groups of devices are fabricated and characterized (Figure 2b). To minimize batch-to-batch variations, the four devices within each comparison group are prepared at the same time. The devices within each group demonstrate a consistent trend as mentioned above. Despite the large standard deviation of the data, Figure 2b still shows the trend of improved device performance when HMDS is inserted at different interfaces. The average current efficiency of the bilateral modified device rises to 17.3 cd/A, which is about 8.2% higher compared with the control device (16.0 cd/A).

The device operational stability was assessed in air by applying a constant current, with the initial brightness being about 10000 cd/m^2^. According to the empirical scaling law widely used for QLED, *L*_0_^*n*^ × *T*_50_ = *C*,^22^ we simulated the lifetime of devices at the initial brightness of 1000 cd/m^2^ when considering the acceleration factor *n* = 1.8 (Figure 2c). Due to the different passivation mechanisms at the various interfaces, the devices experience different decay trends in their operational lifetime. In the early stage, samples without HMDS at QD/ETL interface (Figure 2c gray and red curves) exhibit a near-exponential form of lifetime decay, possibly associated with carrier-filling processes such as defect trapping and reaction-like interfacial processes. When the ETL is modified with HMDS (Figure 2c indigo and sky-blue curves), the luminance decay slows down, showing a quasi-linear relationship, which implies that the interface is effectively passivated, and the associated mechanisms are suppressed. It is worth noting that this modification does not seem to work for the later-stage decay process. In contrast, in the later stage, a rapid linear decay is observed when the HTL/QDs interface is not modified (gray and sky-blue curves). But when HMDS is introduced to the HTL/QDs interface (indigo and red curves), the decay trend becomes gentler. This is partly because HMDS protects the TFB layer from damages caused by the excess electrons accumulating at the interface.

In order to illustrate the specific effect of the HMDS insertion layer on ETL and HTL, respectively, we conducted spectroscopic and electrical characterizations. The time-resolved photoluminescence (TrPL) can demonstrate the impact of the ZnO layer on QDs luminescence. The spectra of the ITO/QDs/ZnO films with and without HMDS modification (Figure 3a) can both be well-fitted by the biexponential equation (Table S1).^23,24^ The exciton lifetime in the QDs increases from 13 to 16 ns when HMDS modification is added at the QDs/ETL interface, suggesting that HMDS can reduce the nonradiative recombination channels caused by ZnO ETL layer (Figures S6 and S7). Also, the I-V curve of the electron-only devices (Figure 3b) shows that the insertion of HMDS at the QDs/ETL interface can effectively boost the electron current, indicating more efficient electron injection and transport capability. Meanwhile, it is observed that the I-V curve of the HMDS-modified sample shows a broader linear ohmic character region, whereas the slope of the I-V curve clearly increases at high voltage in the sample without HMDS. According to the theory of space charge limited conduction, usually this large-slope interval is referred to as the trap-filled limited region, implying the presence of traps in the sample.^25^ So, the HMDS facilitates the carrier transport process and mitigates its impact on quantum dot luminescence by passivating the defects on the ZnO surface.

**Figure 3:**
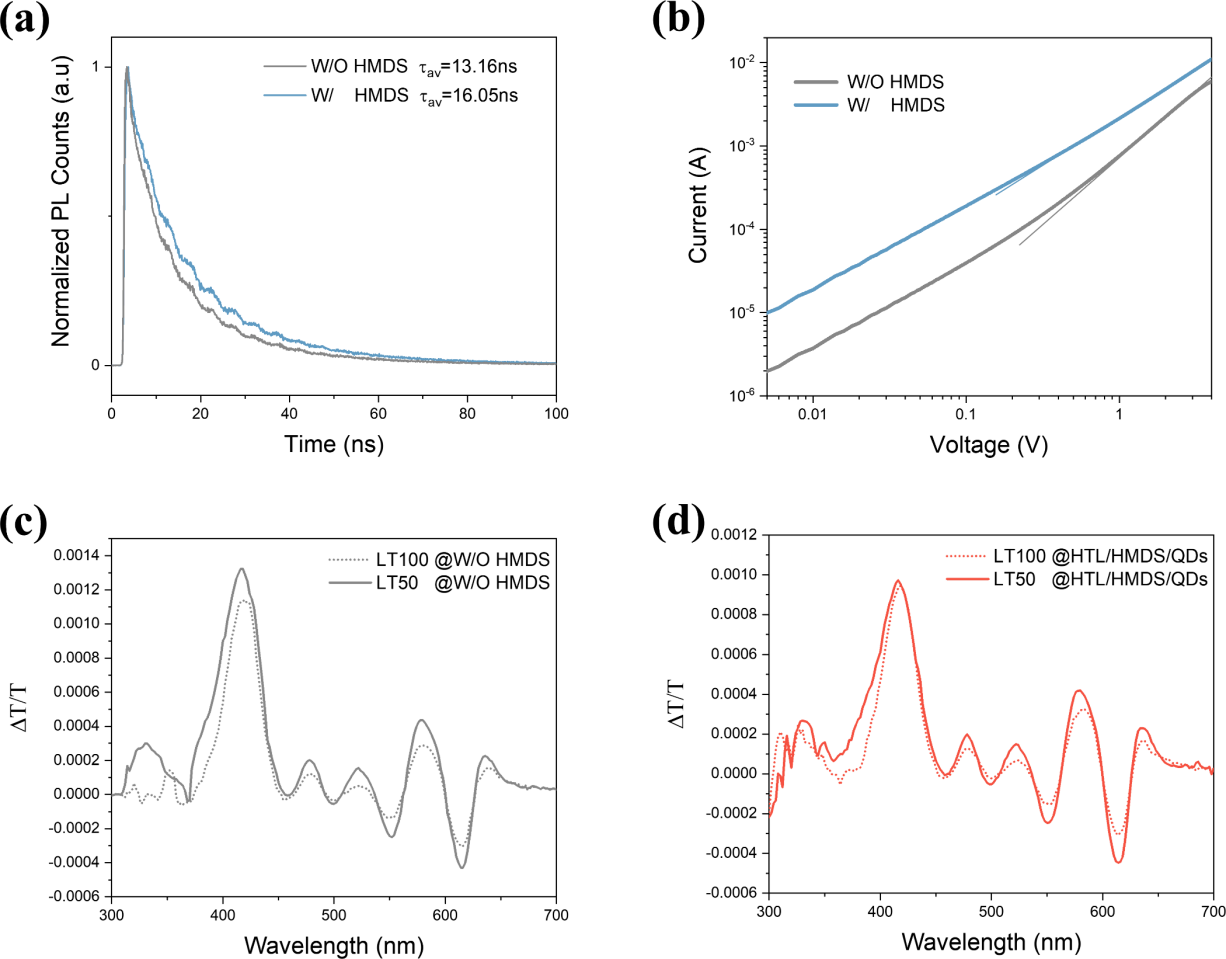
Carrier transport and degradation of devices. **a**, TrPL spectra of ITO/QDs/ZnO films with or without HMDS inserted at QDs/ZnO interface. **b**, Current of electron-only devices (based on structure of ITO/ZnO/QDs /ZnO/Al) with or without HMDS layer at QDs/ZnO interface. **c**, **d**, Electroabsorption spectra of the control device and HTL/HMDS/QDs device before and after aging.

For the HTL side, the HMDS insertion has noticeable beneficial effects on the stability of QLEDs. The HTL degradation is analyzed by electro-absorption spectroscopy (EA) (Figure 3c,d). The EA signal for red QDs in these devices ranges from 450 to 670 nm while that of TFB ranges from 400 to 450 nm, which are related to their energies of optical bandgap.^21^ By comparing the EA signals of LT100 and LT50 devices, we find that they exhibit similar spectral changes, except for the TFB region. The in-phase signal of QDs in the aged sample is relatively strengthened, compared to the fresh sample. This is consistent with the observation that the applied electric field can make a real impact on the property of the emission layer.^26^ In devices without HMDS modification at the HTL/QDs interface (Figures 3c and S8a), there is a marked increase in the 420 nm EA peak of the TFB layer in the aged device compared to the fresh ones, indicating degradation of the TFB layer. However, this alteration is not as pronounced in the devices where the HTL/QDs interface has been modified with the HMDS insertion (Figures 3d and S8b). And after degradation, the PL decay of TFB in the device without HMDS modification is more obvious than that of the device with HMDS modification at the HTL/QDs interface (Figure S9). This suggests that the degradation of the TFB layer is substantially mitigated through the application of an HMDS treatment to the HTL/QDs interface, contributing to enhanced device longevity.

The results above signify that the silane coupling agent HMDS modification at the bilateral interfaces decreased interface states caused by ETL and inhibited degradation of HTL, which can suppress nonradiative recombination and prolong the device’s lifetime. In order to verify the universality of the method above, we repeat the experiments with different types of silane coupling agents. As shown in Figure 4, heptamethyldisilazane (H-silazane), which has seven methyl groups, exhibits the same optimization effect as HMDS. The insertion of H-silazane at different interfaces leads to similar changes in current density and CE. Compared to the control device, CE of the bilateral treatment device is greatly enhanced from 15.2 cd/A to 18.4 cd/A, while the optional lifetime also doubled. Tetramethyldisilazane (T-silazane), which has four methyl groups, was also utilized for the same test and produced consistent results (Figure S10). The findings are consistent with the results above, which also exhibit increased current efficiency (from 15.4 cd/A to 21.8 cd/A) alongside an extended device lifetime.

**Figure 4:**
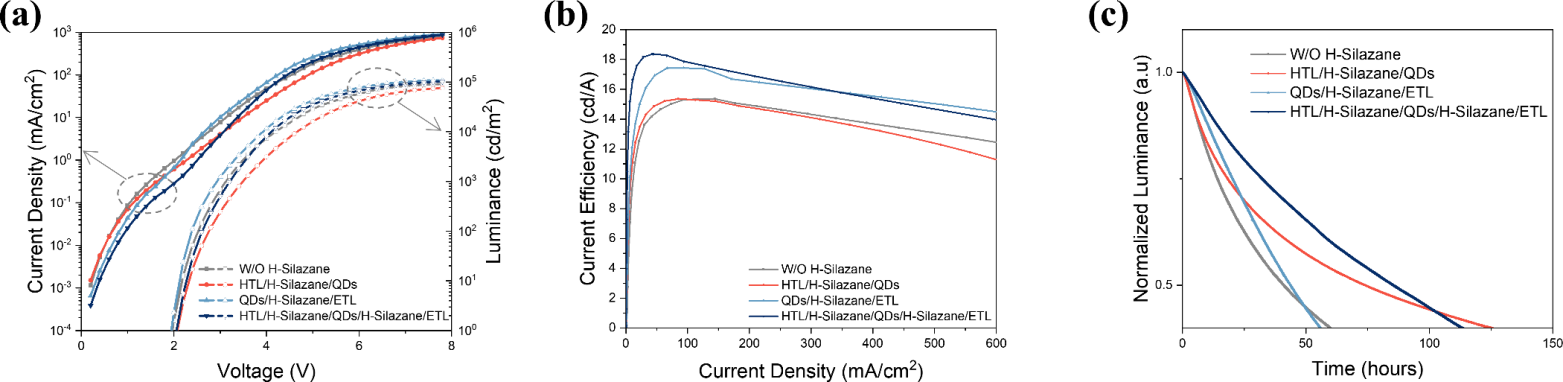
Electrical and aging tests on four groups of devices with different interfacial modifications with heptamethyldisilazane. **a**, Current density versus voltage curves (left) and luminance versus voltage curves (right). **b**, Current efficiency versus current density curves. **c**, Aging test for four groups of devices under a constant current density, whose initial brightness is about 5000 cd/m^2^. The lifetime of devices is simulated at initial brightness of 1000 cd/m^2^ based on the relation *L*_0_^*n*^ × *T*_50_ = *C* (*n* = 1.8).

In summary, based on the principle of saturated adsorption self-limitation, the interface between quantum dots and CTL is modified with a monolayer silane coupling agent, and it is confirmed that this method can efficiently improve the device efficiency and stability. The data show that the function of silane coupling agent at the interface between ETL and HTL is different. The modification on the HTL side mainly delays the degradation of the TFB, thereby improving the long-term stability of the device. While the HMDS on the ZnO ETL side is mainly used to passivate the defects of the ZnO ETL, and the related mechanism has a significant impact on the initial stability of QLED. Although the specific mechanisms affecting device performance are not explicitly identified in this study, in addition to the interface modification strategies mentioned, the preliminary conclusions on affecting the stability of the device may help in understanding the working mechanism of QLED and further improving the stability of the device.

## Supporting Information Available

Experiment details; AFM, SEM images, and TEM-EDS mapping of the control and modified samples; Current of hole-only devices; EL spectra of fresh devices; PL spectra of ZnO films and QDs films; Electro-Absorption spectra of the fresh and aged devices; PL spectra of TFB layer before and after degradation; J-V-L characteristics and aging test of devices with T-silazane insertion; and fitting of TrPL (PDF)
