# Supplementary material for: Atomic Layer-Deposited Silane Coupling Agent for Interface Passivation of Quantum Dot Light-Emitting Diodes
Source: J Phys Chem Lett. 2024 Sep 3;15(36):9233–8. doi: 10.1021/acs.jpclett.4c01974 (PMC11403656; doi:10.1021/acs.jpclett.4c01974)
Supplement: Supplementary file 1 — jz4c01974_si_001.pdf [file jz4c01974_si_001.pdf]

Supporting Information

Atomic Layer-Deposited Silane Coupling Agent for Interface Passivation of Quantum Dot Light-Emitting Diodes

Ting DING, Yin-Man SONG, Meng-Wei WANG, Hang LIU, Jing JIANG, Jin-Cheng XU, Hong-Chao LIU*, Kar-Wei NG*, Shuang-Peng WANG*

Institute of Applied Physics and Materials Engineering, University of Macau, Taipa, Macao SAR 999078, China

Experimental Section

Materials

Zinc acetate dihydrate (98%-101%) was purchased from Alfa Aesar. PEDOT: PSS [Poly(3,4-ethylene dioxythiophene)-poly(styrenesulfonate), LT-100] was purchased from Luminescence Technology Corp. TFB [Poly(9,9-dioctylfluorenyl-2,7-diyl)-alt-(4,4′-(N- (4-butylphenyl), average molecular weight, 140,000 g/mol] was purchased from American Dye Source. Colloidal red QDs CdSe/ZnS were purchased by Guangdong Poly OptoElectronics Co. Ltd. Chlorobenzene (99.8%) and n-Octane (≥99%) were purchased from Thermo Scientific. Ethanol absolute (99.5%) was purchased from Hushi of SCRC. Ethanolamine(≥99.5%), n-Hexane (≥99%) and KOH (99.99%), HMDS (Hexamethyl disilylamine, ≥99%), Heptamethyldisilazane (97%), and 1,1,3,3-Tetramethyl Disilazane (95%) were purchased from Macklin. All chemicals were used directly as received without any further purification.

Synthesis of ZnO nanoparticles

First, 5.5 g Zn $(OAc)_2 \cdot 2H_2O$ was heated at 120 °C for 1 hour under standard air-free conditions to dehydrate. Then 150 mL ethanol absolute was added. The system was in a condensing reflux unit and stirred for 0.5 h at 80 °C to get an evenly dispersed precursor. 2 g of KOH was well dissolved in 20 mL of ethanol and then added drop by drop into the zinc precursor. After stirring for 5 minutes, a white precipitate was obtained after pouring the solution into 450 mL n-hexane. The precipitate was centrifuged and washed sequentially with ethanol and n-hexane. Then the precipitate was placed in a vacuum environment for 2 hours to obtain ZnO nanoparticle powder. Finally, the as-prepared powder was dispersed in ethanol (30 mg/mL) and stabilized with a certain amount of ethanolamine (typically 60 μL per 20 mL solution). The ZnO solution should be filtered using a 0.22 μm nylon filter before use.

Device fabrication

First, the patterned ITO glass substrates were treated with UV-ozone for 15 min to make the ITO surface hydrophilic. Then, the PEDOT: PSS was spin-coated for 40 s at 3000 rpm and baked at 150 °C for 20 min in air. Subsequently, the samples were transferred to a nitrogen-filled glovebox. Then TFB solution (8 mg/ml in chlorobenzene) was spin-coated at 3000 rpm for 40 s and baked at 120 °C for 15 min. After that, the QD solution (20 mg/ml in n-octane) was spin-coated at 3000 rpm, 40 s and baked at 90 °C for 10 min. Then, the ZnO solution (30 mg/ml in ethanol) was spin-coated at 4000 rpm for 40 s and baked at 80 °C for 15 min. Finally, the samples were transferred into a vacuum

evaporation system for Al electrode deposition. The emitting area of the devices is 2×3 mm^2. Acid-free UV-curable resins (Loctite 3335) were used to encapsulate the devices.

Electron-only device fabrication: Cleaned ITO substrates were treated in UV-ozone for 15 min. The ZnO solution was spin-coated on the ITO glass at 3000 rpm for 40 s and annealed at 120 °C for 15 min in a nitrogen-filled glovebox. Then QDs layer was spin-coated. After that, another ZnO layer was spin-coated at 4000 rpm for 40 s and baked at 80 °C for 15 min. Al (100 nm) was sequentially deposited by thermal evaporation in a vacuum deposition chamber. The area of the devices is 0.5 ×0.5 mm^2.

Hole-only device fabrication: Cleaned ITO substrates were treated in UV-ozone for 15 min. The PEDOT: PSS solution was spin-coated onto the ITO glass at 3000 rpm for 40 s and annealed in air at 150°C for 20 min. Then the substrate was transferred into the glovebox. The TFB solution was spin-coated at 3000 rpm for 40 s and baked at 120 °C for 15 min. The QDs layer was then spin cast. TCTA (40 nm), MoO_3 (10 nm) and Al (100 nm) were sequentially deposited by thermal evaporation in a vacuum deposition chamber. The area of the devices is 0.5 ×0.5 mm^2.

Characterization

A combination of a source meter (Keithley 2400) and a luminance meter (Minolta LS-110) was used to measure the current density–voltage–luminance characteristics. The lifetime of devices was tested by a working lifetime system (HMC-8100, Guangzhou Jinghe Equipment Co., Ltd). The TrPL spectra were measured with a Fluorescence

Spectrometer (FS5, Edinburgh) with a 365 nm laser. The capacitance–voltage characterization was measured using a semiconductor device analyzer (B1500A, Keysight) at room temperature, with a modulating frequency of 10k Hz. The surface morphology was characterized by field emission scanning electron microscopy (FE-SEM, Sigma, Carl Zeiss). The cross-sectional morphology and thickness of the devices were conducted by high-resolution transmission electron microscopy (HRTEM, Talos F200X, FEI). The Electro-Absorption measurement is as follows: A parallel beam at different wavelengths produced from the monochromator entered the sample through the ITO side with an incident angle of 45° and is reflected by Al electrode. A calibrated silicon photodetector was used to collect the signals from the reflected beam. The silicon photodetector was connected with a lock-in amplifier (SR830) for low-noise measurement. A sinusoidal bias with a frequency of 1k Hz was superimposed on a negative DC bias of −3 V, AC amplitude of 1 V, which was used to modulate the internal electric field of QLEDs with no carrier injection. The final signal (-ΔT/T) was the ratio of the reflected signals with and without the modulation. For four devices, the spectrum of electro-absorption is obtained by monitoring luminance decaying to 50% of the initial one under constant current density.

Figures and Caption

Figure S1

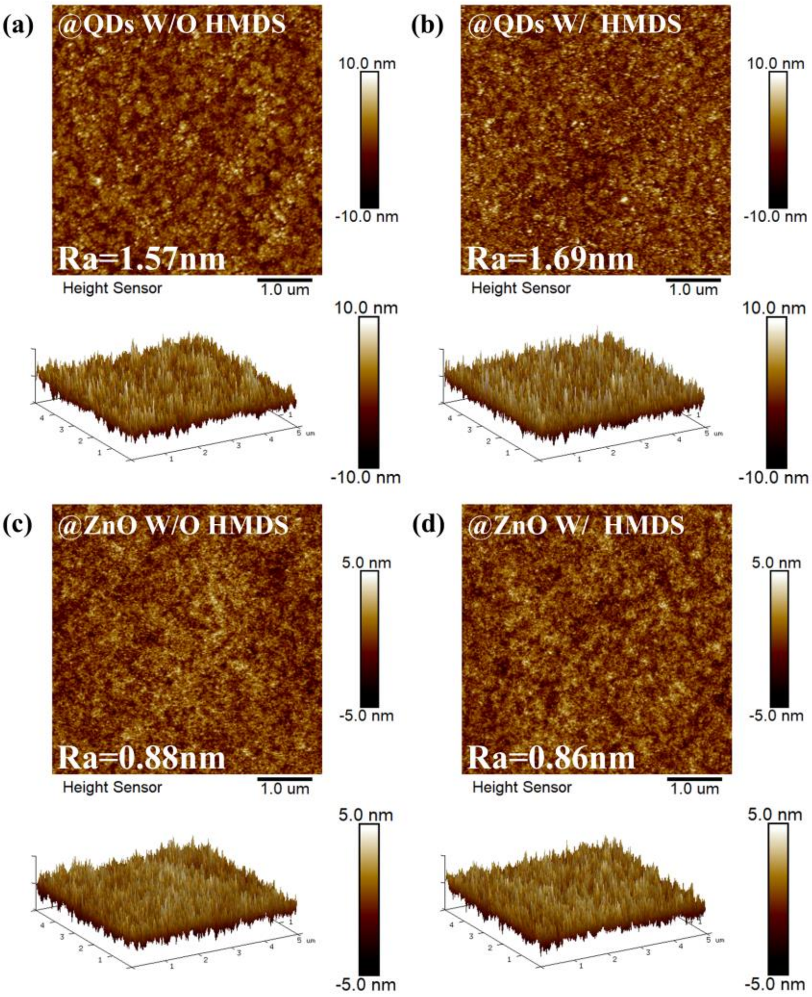

Fig. S1| AFM images before and after modification

AFM images of (a) ITO/PEDOT:PSS/TFB/QDs, (b) ITO/PEDOT:PSS/TFB/HMDS/QDs, (c) ITO/PEDOT:PSS/TFB/QDs/ZnO, and (d) ITO/PEDOT:PSS/TFB/QDs/HMDS/ZnO.

The results indicate that the atmosphere treatment does not affect the morphology of the subsequent spin-coated QDs film and ZnO film, thus introducing negligible effects on the device structure.

Figure S2

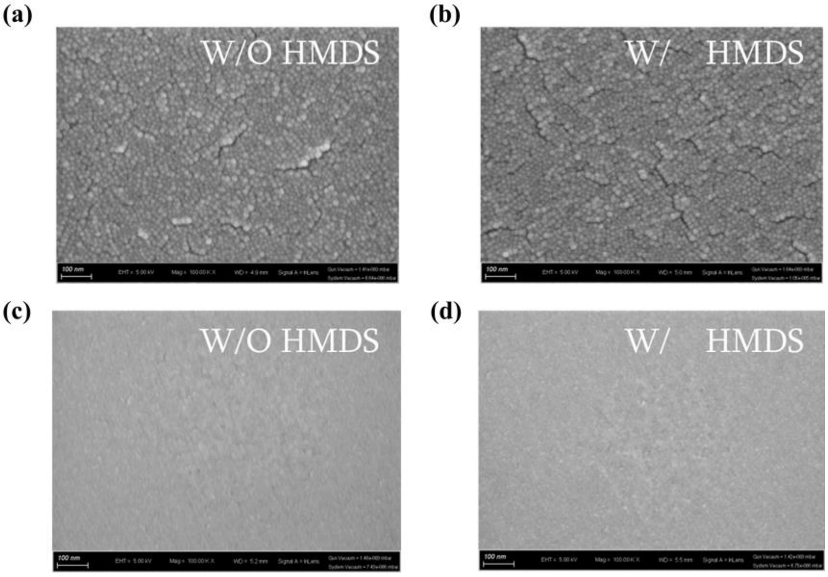

Fig. S2| Morphological changes before and after modification

a, **b**, SEM of ITO/PEDOT:PSS/TFB/QDs film with or without HMDS atmosphere modification at HTL/QDs interface. **c**, **d**, SEM of ITO/PEDOT:PSS/TFB/QDs/ZnO film with or without HMDS atmosphere modification at QDs/ETL interface.

There is no obvious variation in the morphology of QDs and ZnO film after modification of HMDS atmosphere.

Figure S3

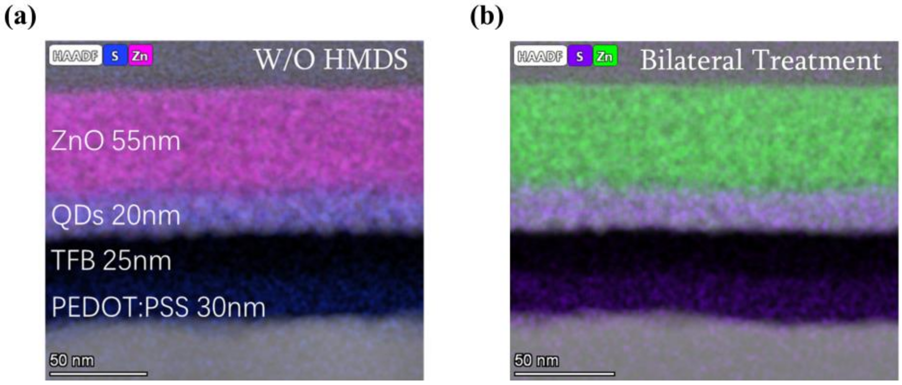

Fig. S3| Cross-section changes before and after modification

a, the Cross-section TEM-EDS mapping analyses of control device. **b**, the Cross-section TEM-EDS mapping analyses of HTL/HMDS/QDs/HMDS/ETL device.

After treatment by HMDS atmosphere, the boundary between layer to layer is still discernible, and there is no obvious change in the thicknesses of the layers.

Figure S4

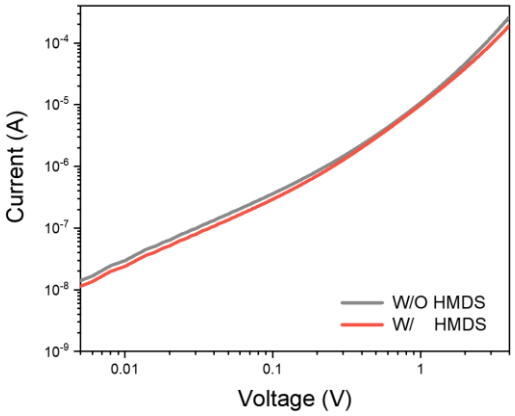

Fig. S4| Current of hole only devices

Current of hole only devices based on structure of ITO/PEDOT:PSS/TFB/QDs/TCTA /MoO_3/Al with and without HMDS at HTL/QDs interface.

With HMDS insertion layer at HTL/QDs interface, the hole current becomes lower, which is caused by insulting property of HMDS.

Figure S5

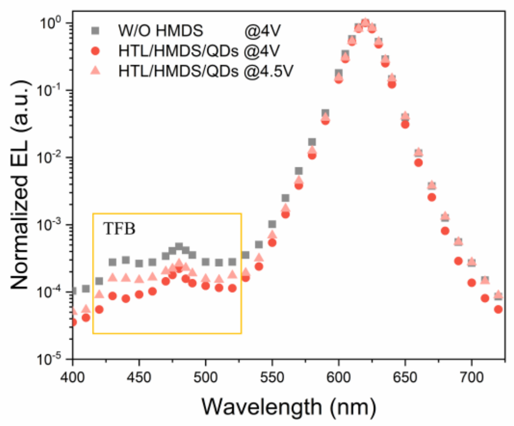

Fig. S5| Normalized EL spectra of fresh devices

The changes of EL spectra of devices (based on the structure of ITO/PEDOT:PSS/TFB /QDs/ZnO/Al) with and without HMDS modification at HTL/QDs interface through single-photon counting technique. The results were normalized by the EL peak of QDs at 620 nm. And the peak around 430 nm and 480 nm are corresponding to TFB emission.

With insertion of HMDS layer, the EL signal of TFB becomes weaker at both two voltages compared with that of control device, indicating that the existence of HMDS layer can indeed suppress the leakage of excess electrons to the HTL side, thus enhancing long-term device lifetime.

Figure S6

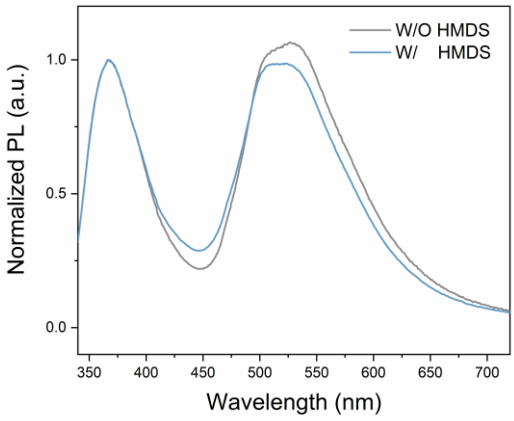

Fig. S6| Normalized PL spectra of ZnO films

The PL spectra of ZnO films (spin-coated in silicon wafer) with and without HMDS atmosphere modification. The results were normalized by the band-edge emission peak of ZnO at 366 nm. And the broad peak centered at 530 nm originates from the ZnO defect states.

With HMDS modification, the PL peak of ZnO film at 530 nm is slightly weakened, which can be attributed to the passivation of ZnO surface defects. This shows that HMDS insertion layer can indeed reduce surface defects in the ZnO film, thus suppressing non-radiative recombination at the QD/ZnO interface.

Figure S7

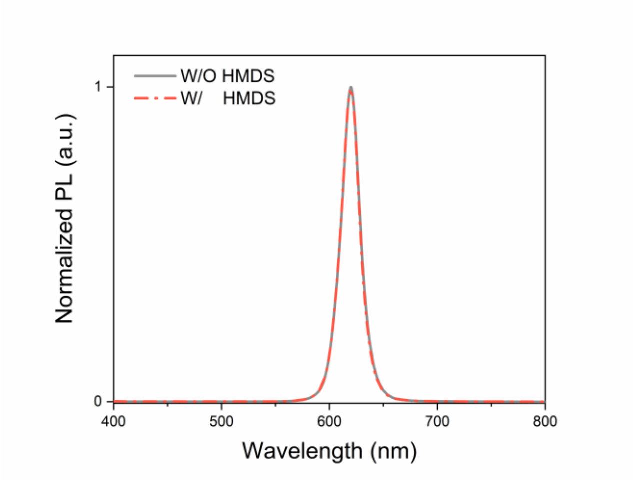

Fig. S7| Normalized PL spectra of QDs films

The PL spectra of QDs films (spin-coated in silicon wafer) with and without HMDS atmosphere modification. The results were normalized by the emission peak of QDs.

As shown in Figure S7, the HMDS layer does not affect the PL property of the QDs layer. The HMDS coating did not cause any change in the peak position or full width at half maximum (FWHM) of the QD. The PLQY of QDs film with HMDS modification is 29.5%, which is the same as the control QDs film (29.6%). So, the HMDS modification does not influence the emission property of the QDs layer. This means that the improved device efficiency is not due to the passivation of QDs.

Figure S8

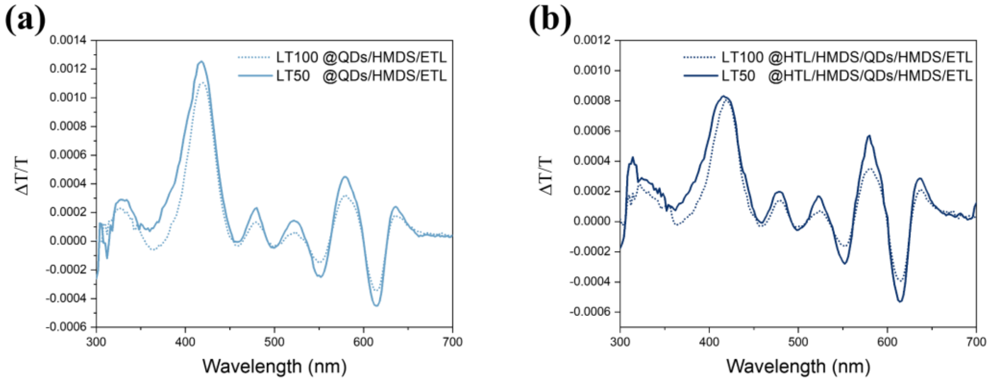

Fig. S8| Electro-absorption spectra

a, **b**, Electro-absorption spectra of QDs/HMDS/ETL device and HTL/HMDS/QDs /HMDS/ETL device before and after aging.

The signal from TFB increases for control device after aging, indicating degradation of the TFB layer. While for HTL/HMDS/QDs/HMDS/ETL device, it remains unchanged, which suggests that the degradation of the TFB layer is substantially suppressed through the application of an HMDS treatment to the HTL/QDs interface.

Figure S9

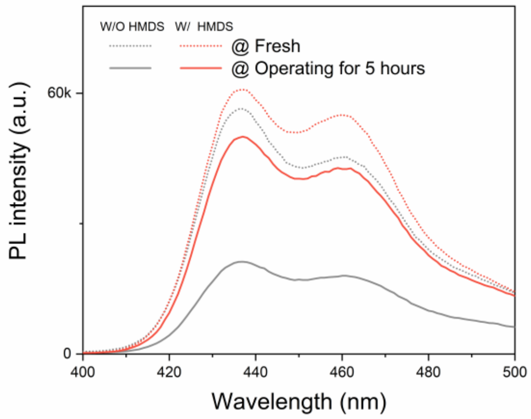

Fig. S9| PL spectra of TFB in devices before and after degradation

The PL of TFB before and after degradation in devices (based on the structure of ITO/PEDOT:PSS/TFB/QDs/ZnO/Al) with and without HMDS modification. The degradation was happened by applying a constant current density of 50 mA/cm^2 to the two devices for 5 hours.

With the HMDS insertion layer, the PL reduction of TFB after degradation is decreased, which means the degradation of TFB is suppressed. This shows the insertion of HMDS layer can indeed help to prolong the device lifetime by lessening the degradation of TFB.

Figure S10

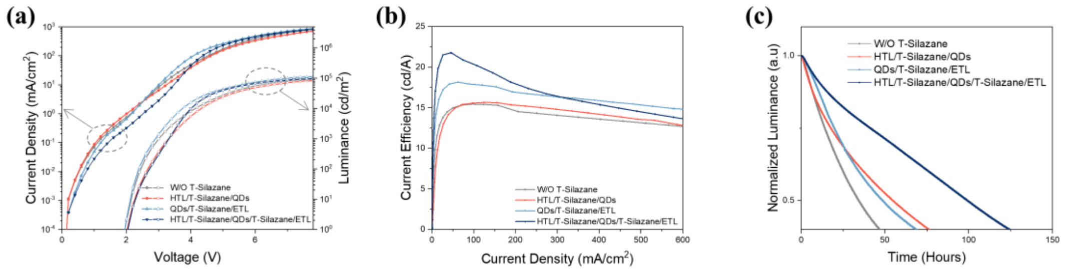

Fig. S10| Electrical tests and Aging test on devices modified by Tetramethyldisilazane (T-silazane) atmosphere

a, Current density versus voltage curves(left) and luminance versus voltage curves(right). **b**, Current efficiency versus current density curves. **c**, Aging test for four groups of devices under a constant current density with initial brightness of about 5000 cd/m^2, from which we simulated the lifetime of devices at initial brightness of 1000 cd/m^2 based on the relation $L_0^n \times T_{50} = C$. (n = 1.8)

The results are consistent with what we observed on devices modified by HMDS atmosphere.

Table S1

	A_1	τ_1 (ns)	A_2	τ_2 (ns)	τ_{av}
W/O HMDS	0.40235	5.18249	0.57753	15.07267	13.16
W/ HMDS	0.40928	7.38084	0.55783	18.58128	16.05

Table. S1| Fitting results of TrPL

The bi-exponential decay function is used to fit curves of the TrPL spectra of ITO/QDs/ZnO film with or without HMDS at QDs/ZnO interface.

$$y = y_0 + A_1 * \exp [- (x - x_0) / \tau_1] + A_2 * \exp [- (x - x_0) / \tau_2]$$

where τ_i and A_i are the corresponding decay time and wights, respectively. Then we can calculate average exciton lifetime by equation

$$\tau_{av} = (A_1 \tau_1^2 + A_2 \tau_2^2) / (A_1 \tau_1 + A_2 \tau_2)$$
